# Gene × Gene interaction between *MnSOD *and *GPX-1 *and breast cancer risk: a nested case-control study

**DOI:** 10.1186/1471-2407-6-217

**Published:** 2006-08-31

**Authors:** David G Cox, Rulla M Tamimi, David J Hunter

**Affiliations:** 1 Program in Molecular and Genetic Epidemiology, Epidemiology Department, Harvard School of Public Health, 677 Huntington Ave., Boston, MA 02115, USA; 2 Channing Laboratory, Department of Medicine, Brigham and Women's Hospital, 181 Longwood Ave., Boston, MA 02115, USA; 3 Epidemiology Department, Harvard School of Public Health, 677 Huntington Ave., Boston, MA 02115, USA

## Abstract

**Background:**

Germ-line mutations in genes such as *BRCA1*, *BRCA2*, and *ATM *can cause a substantial increase in risk of breast cancer. However, these mutations are rare in the general population, and account for little of the incidence of sporadic breast cancer in the general population. Therefore, research has been focused on examining associations between common polymorphisms and breast cancer risk. To date, few associations have been described. This has led to the hypothesis that breast cancer is a complex disease, whereby a constellation of very low penetrance alleles need to be carried to present a risk phenotype. Polymorphisms in the manganese superoxide dismutase (*MnSOD*) and glutathione peroxidase (*GPX-1*) genes have been proposed as low penetrance alleles, and have not been clearly associated with breast cancer. We investigated whether variants at both polymorphisms, while not independently associated with breast cancer risk, could influence breast cancer risk when considered together.

**Methods:**

A case-control study nested within the Nurses' Health Study was performed comparing 1262 women diagnosed with breast cancer to 1533 disease free women. The *MnSOD *(Val16Ala, rs1799725) and *GPX-1 *(Pro198Leu, rs1050450) were genotyped via TaqMan assay. Disease risk was evaluated using logistic regression.

**Results:**

While neither allele alone shows any change in breast cancer risk, an increase in the risk of breast cancer (OR 1.87, 95% CI 1.09 – 3.19) is observed in individuals who carry both the Ala16Ala genotype of *MnSOD *and the Leu198Leu genotype of *GPX-1*.

**Conclusion:**

Polymorphisms in the *GPX-1 *and *MnSOD *genes are associated with an increased risk of breast cancer.

## Background

Both manganese superoxide dismutase and glutathione peroxidase act as antioxidant enzymes [1,2]. Non-synonymous polymorphisms in both genes have been shown to reduce their effectiveness at removing oxidative species from cells [3,4]. Oxidative stress has been hypothesized to be involved in breast cancer risk, and there is evidence supporting a modest inverse association between levels of antioxidants and breast cancer risk [5]. However, polymorphisms in these two genes do not individually alter breast cancer risk [6,7].

Few polymorphisms have shown statistically significant associations with breast cancer risk to date. One explanation for this lack of association could be that common polymorphisms do not have a large enough effect on the function of any particular gene to be responsible for cancer development alone. Rather, polymorphisms in several biologically related genes which slightly modify the function of each individual gene, may combine to explain the genetic component of breast cancer risk. Additionally, lifestyle factors could also play a role in disease etiology, either independently or in combination with genetic variants.

We have previously genotyped non-synonymous polymorphisms in the *MnSOD *(Val16Ala, rs4880) and *GPX-1 *(Pro198Leu, rs1050450) genes, and found no association between these individual polymorphisms and breast cancer risk [6,7]. However, given that both genes act as antioxidants, we hypothesized that being homozygous for the 198Leu allele of *GPX-1 *which is less responsive to selenium [3], and the 16Ala allele of *MnSOD *which is associated with increased urinary DNA adduct levels [4] increases breast cancer risk.

## Methods

Genotyping assays for the *MnSOD *(Val16Ala, rs1799725) and *GPX-1 *(Pro198Leu, rs1050450) polymorphisms were performed by the 5' nuclease assay (TaqMan) on the ABI Prism 7900HT Sequence Detection System (Applied Biosystems, Foster City, CA). TaqMan primers, probes, and conditions for genotyping assays are available on request from the authors. Our study included a total of 1262 incident breast cancer cases diagnosed after blood draw up to June 1, 2000, and 1533 matched controls, all drawn from 32,826 women in the Nurses' Health Study who gave a blood sample in 1989–90. Controls were randomly selected participants who were free of diagnosed cancer (except non-melanoma skin cancer), and matched to cases based on age, menopausal status, recent post-menopausal hormone use, and time, day, and month of blood collection. A detailed description of this study population has been previously reported [8]. Approximately 95% of the samples were successfully genotyped for each polymorphism individually; samples that failed genotyping for both polymorphisms were removed from the analyses. Internal blinded quality control samples (49 sets of replicate samples, including from 2 to 9 replicates) showed 100% concordance.

We used SAS v9.1 (SAS Institute, Cary, NC) for all statistical analyses. Odds ratios (OR) and 95% confidence intervals (CI) were calculated using unconditional logistic regression, controlling for matching factors, age at menopause, age at menarche, age at first birth and parity, body mass index at age 18, weight gain since age 18, history of benign breast disease, and family history of breast cancer using PROC LOGISTIC. Interactions were tested by likelihood ratio tests comparing the model with main effects for each polymorphism to the model including indicator variables for the cross-tabulation of the two polymorphisms. All p-values reported are two-sided. Power calculations were carried out using Quanto [9]. This study was approved by the Institutional Review Board of Brigham and Women's Hospital (protocol # 1999-P-001718).

## Results and discussion

Table 1 shows the cross-tabulation of both the *MnSOD *and *GPX-1 *genotypes and breast cancer risk. The distribution of the combined genotypes shown is not statistically different from that expected, given the respective genotype frequencies (Chi-squared = 3.14 with 1 d.f p = 0.08). Neither of these two SNPs individually increases breast cancer risk. Given that these two genes are active on the pathway of detoxification of reactive oxygen species (ROS) from O^- ^to H_2_O_2 _(*MnSOD*), and further to H_2_O (*GPX-1*) and these polymorphisms influence the efficiency of this detoxification, we hypothesized that risk could be detected when the two SNPs are combined. In these analyses, the combination of the two polymorphisms is associated with significantly increased breast cancer risk. Individuals homozygous for the Ala16 allele of *MnSOD *and Leu198 allele of *GPX-1 *have a 1.87 fold increase in breast cancer risk compared to Val16 and Pro198 carriers, and the p-value for the interaction between these genotypes is 0.03. Further *in vitro *or *in vivo *work should be carried out to examine the differences in ROS levels in cells containing variant *GPX-1 *and *MnSOD *alleles.

To date, few such interactions have been detected and reported in the literature. Of major concern to studies examining the interaction between multiple exposures (either gene × gene or gene × environment) on disease risk is study size. In order to have sufficient power to detect such associations, large data sets are necessary. Our study has 80% power to detect an odds ratio of 1.85 for an interaction between the recessive models for two polymorphisms such as those reported here, where neither polymorphism alone increases risk. In the present analyses, we benefit from the relatively high prevalence of the two polymorphisms. If the prevalence of one or both polymorphisms was lower, very large studies would be needed to detect an interaction similar to the one we observed. Of obvious interest would be to examine more genes along the same pathway, as well as non-genetic variables such as plasma antioxidant levels or lifestyle factors which affect oxidative stress such as cigarette smoking, and consumption of alcohol, as well as dietary antioxidants or supplements. However, including further variables would necessitate even larger sample size. Future work on polymorphisms in genes along biologically related pathways, and the inclusion of gene × environment interactions, will necessitate further development of statistical methods. Currently accepted methods to model the association between large numbers of variables (both genetic and non-genetic) and disease risk are plagued with problems of sparse data, as well as model choice and uncertainty. When methods robust to these problems are developed, further examination of the antioxidant activity pathway via multivariate analyses of gene × gene and gene × environment interactions can be examined.

## Conclusion

Polymorphisms in the *GPX-1 *and *MnSOD *genes are associated with an increased risk of breast cancer.

## Abbreviations

MnSOD: Manganese superoxide dismutase

GPX-1: Glutathione peroxidase 1

OR: Odds Ratio

CI: Confidence Interval

## Competing interests

The author(s) declare that they have no competing interests.

## Authors' contributions

DGC performed statistical analyses and wrote the manuscript with RMT. DJH designed and obtained funding for the study, and provided editorial support. All authors have read and approved the final manuscript.

## Pre-publication history

The pre-publication history for this paper can be accessed here:



## Figures and Tables

**Table 1:** Interaction between GPX1 Pro198Leu and MnSOD Val16Ala polymorphisms and breast cancer risk in the Nurses' Health Study

**Genotype**	Cases (%)*	Controls (%)*	OR (95% CI)**
Pro198 carrier and Val16 carrier	771 (67.0)	997 (67.4)	1.00 (Ref.)
Leu198Leu and Val16 carrier	90 (7.8)	124 (8.4)	0.94 (0.69 – 1.27)
Pro198 carrier and Ala16Ala	255 (22.2)	331 (22.4)	1.01 (0.83 – 1.23)
Leu198Leu and Ala16Ala	35 (3.0)	28 (1.9)	1.87 (1.09 – 3.19)

* Samples failing genotyping for both assays were removed from the analyses

** Unconditional logistic regression, controlled for fasting status, date and time of blood draw, age at blood draw (5 year categories), menopausal status, recent post-menopausal hormone use at blood draw, age at menopause, age at menarche, body mass index at age 18, weight gain since age 18, age at fist birth/parity, history of benign breast disease, and family history of breast cancer. Interaction p-value = 0.03.

## References

[B1] Briehl MM, Baker AF, Siemankowski LM, Morreale J (1997). Modulation of antioxidant defenses during apoptosis. Oncol Res.

[B2] Arthur JR (2000). The glutathione peroxidases. Cell Mol Life Sci.

[B3] Hu YJ, Diamond AM (2003). Role of glutathione peroxidase 1 in breast cancer: loss of heterozygosity and allelic differences in the response to selenium. Cancer Res.

[B4] Hong YC, Lee KH, Yi CH, Ha EH, Christiani DC (2002). Genetic susceptibility of term pregnant women to oxidative damage. Toxicol Lett.

[B5] Tamimi RM, Hankinson SE, Campos H, Spiegelman D, Zhang S, Colditz GA, Willett WC, Hunter DJ (2005). Plasma carotenoids, retinol, and tocopherols and risk of breast cancer. Am J Epidemiol.

[B6] Tamimi RM, Hankinson SE, Spiegelman D, Colditz GA, Hunter DJ (2004). Manganese superoxide dismutase polymorphism, plasma antioxidants, cigarette smoking, and risk of breast cancer. Cancer Epidemiol Biomarkers Prev.

[B7] Cox DG, Hankinson SE, Kraft P, Hunter DJ (2004). No association between GPX1 Pro198Leu and breast cancer risk. Cancer Epidemiol Biomarkers Prev.

[B8] De Vivo I, Hankinson SE, Colditz GA, Hunter DJ (2003). A functional polymorphism in the progesterone receptor gene is associated with an increase in breast cancer risk. Cancer Res.

[B9] Gauderman WJ (2002). Sample size requirements for matched case-control studies of gene-environment interaction. Stat Med.

